# Localisation-based imaging of malarial antigens during erythrocyte entry reaffirms a role for AMA1 but not MTRAP in invasion

**DOI:** 10.1242/jcs.177741

**Published:** 2016-01-01

**Authors:** David T. Riglar, Lachlan Whitehead, Alan F. Cowman, Kelly L. Rogers, Jake Baum

**Affiliations:** 1 The Walter and Eliza Hall Institute of Medical Research, Melbourne, Victoria, 3052, Australia; 2 Department of Medical Biology, University of Melbourne, Victoria, 3050, Melbourne, Australia; 3 Department of Life Sciences, Imperial College, London SW7 2AZ, UK

**Keywords:** *Plasmodium falciparum*, Merozoite, Erythrocyte invasion, Tight junction, Deconvolution

## Abstract

Microscopy-based localisation of proteins during malaria parasite (*Plasmodium*) invasion of the erythrocyte is widely used for tentative assignment of protein function. To date, however, imaging has been limited by the rarity of invasion events and the poor resolution available, given the micron size of the parasite, which leads to a lack of quantitative measures for definitive localisation. Here, using computational image analysis we have attempted to assign relative protein localisation during invasion using wide-field deconvolution microscopy. By incorporating three-dimensional information we present a detailed assessment of known parasite effectors predicted to function during entry but as yet untested or for which data are equivocal. Our method, termed longitudinal intensity profiling, resolves confusion surrounding the localisation of apical membrane antigen 1 (AMA1) at the merozoite–erythrocyte junction and predicts that the merozoite thrombospondin-related anonymous protein (MTRAP) is unlikely to play a direct role in the mechanics of entry, an observation supported with additional biochemical evidence. This approach sets a benchmark for imaging of complex micron-scale events and cautions against simplistic interpretations of small numbers of representative images for the assignment of protein function or prioritisation of candidates as therapeutic targets.

## Abstract (precis)

**Highlighted Article:** Here we develop a high-definition imaging approach to dissect and assign function to proteins involved in the rapid process of malaria parasite invasion of the human erythrocyte.

## INTRODUCTION

In the biological sciences, microscopy is generally used to translate information contained within a tissue or cell specimen into a more useful format, such as a processed digital image. Although the degradation of data due to imaging and image analysis is generally well understood and accepted (North, 2006), the loss of information resulting from image selection and presentation is frequently ignored. Certainly, one of the key strengths of high-throughput automated and quantitative imaging studies is that they circumvent these issues and allow the presentation of large quantities of data without interference from direct bias (Zhan et al., 2015). Many imaging workflows, however, are not amenable to such approaches. Imaging of *Plasmodium falciparum* merozoites caught during invasion of the human erythrocyte (Boyle et al., 2010b; Riglar et al., 2011) is one such example.

The blood stage merozoite is one of the smallest eukaryotic cells. Responsible for setting up the blood stages of infection [and, therefore, for the entirety of malaria disease pathology (White et al., 2014)], each merozoite measures only 1–2 μm in length, with a cellular role almost entirely geared towards erythrocyte invasion (Bannister and Mitchell, 2009). The process of invasion [reviewed by Cowman et al. (2012)] commences with a weak attachment between multiple merozoite surface proteins (MSPs) and the erythrocyte. This initial contact between the merozoite and erythrocyte surfaces induces physical changes in the target erythrocyte membrane, possibly due to reorganisation of the erythrocyte cytoskeleton (Zuccala and Baum, 2011). This is followed by reorientation of the merozoite to its apical pole, bringing the core invasion machinery at the parasite's apex in contact with the erythrocyte membrane (Dvorak et al., 1975). The apical complex at this pole contains specialised organelles termed micronemes and rhoptries that secrete protein and lipids during invasion (Cowman et al., 2012). Following an as yet unknown trigger signalling commitment to invasion, the merozoite becomes irreversibly attached to the erythrocyte and initiates a cascade of cellular and molecular events that guide the invasion process through to completion (Hanssen et al., 2013; Riglar et al., 2011; Weiss et al., 2015).

Chief among these interactions is the establishment of the tight or moving junction (Aikawa et al., 1978; Bannister et al., 1975), a molecular aperture in the red cell through which the parasite passes en route to infection. The junction, acting as the key organising nexus for invasion, is thought to correspond to a key molecular interaction between two classes of parasite protein: the rhoptry neck proteins (RONs), which are embedded in or under the target erythrocyte membrane (although parasite derived); and the micronemal protein apical membrane antigen 1 (AMA1), which is present on the merozoite surface at the time of invasion (Riglar et al., 2011). Evidence from both *P. falciparum* and a closely related apicomplexan parasite, *Toxoplasma* *gondii*, demonstrates that AMA1 binds an extracellular loop of RON2 through a deep molecular interaction (Lamarque et al., 2011; Srinivasan et al., 2011; Tonkin et al., 2011; Tyler and Boothroyd, 2011). This link has led to the hypothesis that the tight junction is the point of traction for the merozoite (and *T. gondii* tachyzoite) during invasion, against which (either directly or indirectly) the forces of the internal parasite actomyosin motor (or gliding motor) leverage to drive the parasite into the host cell (Angrisano et al., 2012; Bichet et al., 2014). The motor itself is housed in the outer pellicle of the merozoite and is formed between a short single-headed class XIV myosin interacting with dynamic, although poorly understood, filaments of actin (Baum et al., 2006a). As invasion progresses, various MSPs are differentially proteolytically cleaved from the surface (Bannister et al., 1975; Boyle et al., 2014; O'Donnell et al., 2006). In addition, a largely parasite-derived vacuole surrounding the invading merozoite is established; this structure, which is called the parasitophorous vacuolar membrane (PVM), is thought to be composed of a combination of parasite and host cell lipids (Lingelbach and Joiner, 1998). Upon completion of invasion, the erythrocyte membrane and PVM seal and the tight junction disintegrates (Riglar et al., 2011, 2013).

Once the events of invasion are initiated, the tight junction is one of the clearest reference points for determining the organisation of molecular events at the site of entry (Riglar et al., 2011; Zuccala et al., 2012). However, the absolute size of the junction, at ∼1 μm in diameter, presents a significant challenge for assigning protein presence, absence or colocalisation with its small structure. To overcome this we have previously used wide-field deconvolution and three-dimensional structured illumination (3D SIM) ‘super resolution’ microscopy methods to observe the 3D distribution of protein labelling during merozoite invasion (Riglar et al., 2011; Zuccala et al., 2012). Even with these advances, analyses have most often been limited to the presentation of a small number of representative images. Given that significant variability between individual parasites is often seen (Zuccala et al., 2012), the presentation of low numbers of images combined with the size of the merozoite in comparison to microscopy resolution limits presents a major challenge when attempting to assign an absolute distribution of a protein through time.

Given these challenges we sought to adapt our methods for analysing images of invading merozoites (Boyle et al., 2010b; Riglar et al., 2011), developing a computational workflow towards a more quantitative and unbiased determination of protein distribution during the process of merozoite invasion. In particular, we focussed on the longitudinal distribution of proteins with respect to the tight junction as the merozoite enters the erythrocyte and on the variability shown across individual parasites.

## RESULTS

### A workflow towards the unbiased localisation of proteins at the merozoite–erythrocyte tight junction

**Fig. 1.**
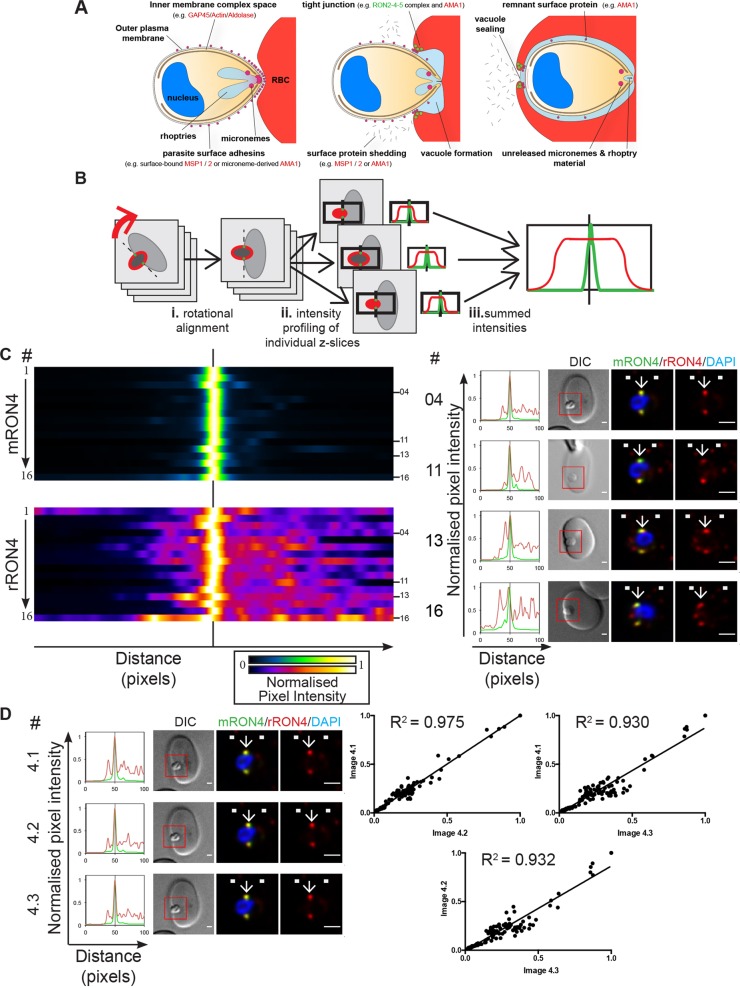
**A workflow for longitudinal intensity profiling of proteins during merozoite invasion.** (A) Schematic of merozoite invasion showing stages of invasion, tight junction formation, micronemal and rhoptry release and surface protein shedding, with some of the key proteins involved. (B) To analyse the localisation of proteins during invasion with respect to the tight junction in an unbiased manner, invading merozoites labelled for RON4 and with another antibody of interest were chosen for imaging based on RON4 labelling and orientation and imaged by widefield fluorescence deconvolution microscopy. (i) Images were rotated so as to align invading left-to-right along the *x*-axis of the image and (ii) were then analysed using a max intensity plot profile along the long axis of the merozoite, centred to the brightest point in the RON4 labelling channel. (iii) Pixel intensity values were then summed to provide an average longitudinal distribution for each merozoite. (C) Heatmaps (left), normalised intensity plots (middle) and single-slice images (right) of four example merozoites (04, 11, 13 and 16) for the longitudinal intensity profiling of mouse RON4 (mRON4; green) versus rabbit RON4 (rRON4; red) antibody labelling, demonstrating the validity of this technique. See also Fig. S1A. (D) Confirmation of workflow accuracy. Three independent replicate images of a single invading merozoite (left) were analysed for correlation by plotting corresponding values from the normalised longitudinal intensity profiles against one another (right). The boxed regions in DIC images are shown magnified to the right. Arrows indicate the position of the RON4-labelled tight junction. White boxes denote approximate front and back position of merozoite as determined in the DIC channel. Scale bars: 1 μm.

Recognising the need for a robust and reproducible means of localising proteins during the process of malaria parasite entry into the human erythrocyte (Fig. 1A), we developed a workflow (longitudinal intensity profiling) of image capture and processing to localise proteins to the merozoite–erythrocyte tight junction with respect to the junctional marker RON4 (see Materials and Methods and Fig. 1B). Although variations in the extent of invasion still prohibit wholesale quantification across multiple parasites (except perhaps for the region directly within the RON4 tight junction ring), the realignment provided by rotation and skew correcting steps in this workflow should allow unprecedented reliability in evaluating large numbers of merozoite invasion events.

As a first test to explore the reproducibility and utility of this workflow, invading merozoites were labelled using rabbit antiserum and mouse monoclonal antibodies, both raised against RON4 (Richard et al., 2010) [Fig. 1C; Fig. S1A; total number of merozoites analysed (*n*)=16]. A single, clear RON4 peak was present in almost every line profile, particularly using the monoclonal, confirming the successful realignment of data in the *z*-direction. Peak intensity in both channels was consistently found at the same point (e.g. Fig. 1C right panel, example parasites 4 and 11). As previously observed (Riglar et al., 2011), background fluorescence was noticeable in many samples labelled using rabbit RON4 antisera, both within the parasite and within the erythrocyte, visible as a higher normalised baseline labelling than that of the monoclonal and in common spurious minor peaks within intensity profiles (e.g. Fig. 1C right panel, example parasites 13 and 16). In all cases the intensity profile matched features that were visible within the 3D image stacks (data not shown), although these were not always apparent in individual slices, such as those depicted in the example merozoite figures displayed in the right panel of Fig. 1C.

Of note, taking an individual invading merozoite through the analysis pipeline three separate times (Fig. 1D) gave very similar results (pairwise R^2^ values of linear regressions for correlations between corresponding values of normalised longitudinal intensity profiles between image pairs of 0.975, 0.930 and 0.932), suggesting that the workflow is robust and provides a reliable means for assessing protein localisation during invasion. The initial results also supported the preferential use of the mouse RON4 monoclonal as a control label for the tight junction in further assays.

### Analysis of AMA1 during invasion identifies the presence of masked epitopes in samples

Having established a reliable computational workflow for longitudinal intensity profiling, we next applied this method to a second key component of tight-junction-dependent invasion: AMA1 (Tyler et al., 2011). Although a large body of literature points to this protein playing a crucial role at the tight junction, recent publications have called into question whether it is essential during malaria parasite (*Plasmodium*) invasion (Bargieri et al., 2013; Yap et al., 2014), as well as during that of the related apicomplexan parasite *T.* *gondii* (Bargieri et al., 2013; Lamarque et al., 2014). Some of this discussion has focussed on AMA1 localisation at the tight junction; for example, its general absence from this site during *T. gondii* tachyzoite invasion of fibroblasts (Giovannini et al., 2011). Our own imaging of 20 invading *P. falciparum* merozoites co-labelled with the RON4 monoclonal antibody demonstrated that AMA1 is very much present and, most often, concentrated at the tight junction during invasion (Riglar et al., 2011).

**Fig. 2.**
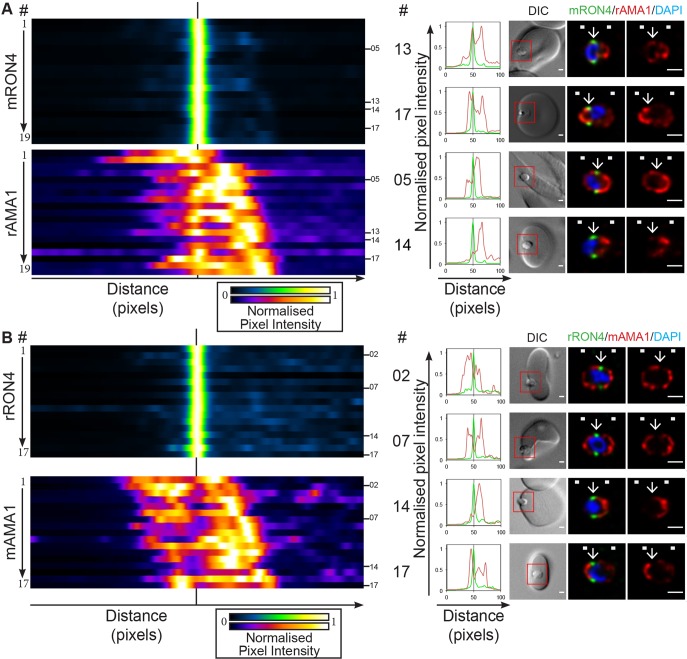
**Longitudinal intensity profiling demonstrates antibody-specific variation in AMA1 detection at the tight junction.** Heatmaps, normalised intensity plots and single-slice images of example merozoites for the longitudinal intensity profiling of (A) mRON4 (green) versus rAMA1 (red) or of (B) rRON4 (green) versus mAMA1 (red) antibody labelling. The boxed regions in DIC images are shown magnified to the right. Arrows indicate the position of the RON4-labelled tight junction. White boxes denote approximate front and back position of merozoite as determined in the DIC channel. See also Fig. S1B,C. Scale bars: 1 μm.

To readdress the population-level variability of AMA1 localisation during *P. falciparum* merozoite invasion using longitudinal intensity profiling, invading merozoites were co-labelled with either the RON4 monoclonal (Richard et al., 2010) and rabbit AMA1 antiserum (Healer et al., 2002) or rabbit RON4 antiserum (Richard et al., 2010) with the well-characterised AMA1 1F9 monoclonal antibodies (Coley et al., 2001). Consistent with our previous results (Riglar et al., 2011), rabbit AMA1 antiserum labelled a portion of AMA1 localised directly within the RON4 annulus in all parasites imaged (Fig. 2A; Fig. S1B; *n*=19). This junctional AMA1 population varied in its relative intensity compared with global AMA1 labelling, which regularly included apical and broad surface localisations. Importantly, however, even the lowest level of junctional AMA1 labelling was appreciably higher than background labelling levels (e.g. Fig. 2A, example 14).

In contrast to polyclonal labelling, AMA1 1F9 monoclonal antibodies showed a clear absence of signal within the RON4-demarked tight junction, evident as local minima in intensity profiles across the parasites imaged (Fig. 2B; Fig. S1C; *n*=17). AMA1 1F9 antibodies bind the hydrophobic cleft of the AMA1 molecule (Coley et al., 2007), the same groove with which RON2 interacts (Lamarque et al., 2011; Tyler and Boothroyd, 2011). When viewed together, these results suggest that AMA1 is indeed present at the tight junction of invading *P. falciparum* merozoites, with micronemal and surface populations also regularly present, but that selection of the appropriate immune label is critical. The 1F9 epitope is clearly masked by interactions occurring at the tight junction, most likely with RON2, under the fixation and permeabilisation conditions used. This is likely to explain the absence of labelling seen in previous studies with *T. gondii* as the result of epitope masking (Giovannini et al., 2011) and highlights the importance of using multiple antibodies to assign protein localisation, in particular polyclonal antibodies with multiple epitopes. Various attempts to expose masked epitopes, including heat, organic solvent based-permeabilisation (e.g. methanol or acetone) and other detergents (e.g. saponin) were unsuccessful. This remains an important limitation for the imaging of invading *P. falciparum* merozoites using our current method.

### Actin and aldolase localise in a manner consistent with the motor model during merozoite invasion

The current model for actomyosin motor involvement during invasion, although the subject of various conflicting genetic deletion data (Andenmatten et al., 2013; Drewry and Sibley, 2015; Egarter et al., 2014), remains largely untested in *P. falciparum* merozoite invasion. We, therefore, used the longitudinal intensity profiling workflow to systematically dissect the localisation of several key components of the parasite's actomyosin motor during merozoite invasion.

**Fig. 3.**
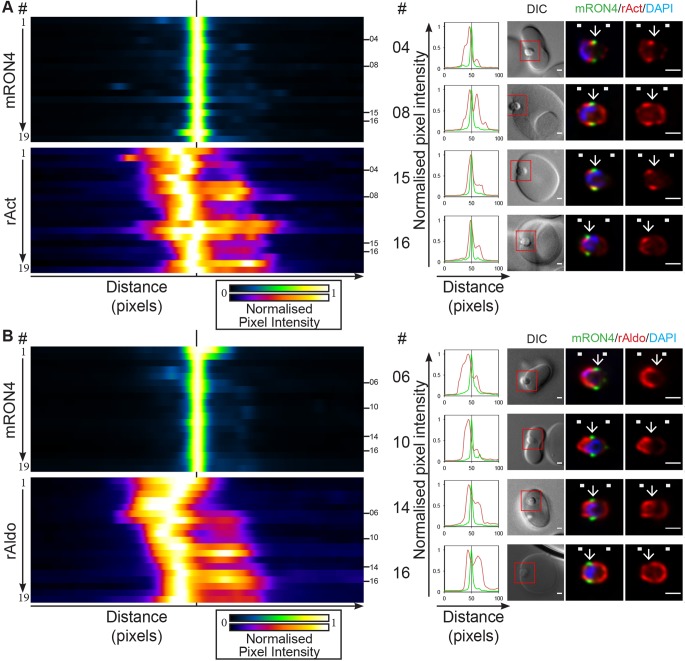
**Longitudinal intensity profiling localises actin and aldolase towards the rear of the invading merozoite tight junction.** Heatmaps, normalised intensity plots and single-slice images of example merozoites for the longitudinal intensity profiling of mRON4 (green) versus (A) rabbit actin (rAct; red) or (B) rabbit aldolase (rAldo; red) antibody labelling. The boxed regions in DIC images are shown magnified to the right. Arrows indicate the position of the RON4-labelled tight junction. White boxes denote approximate front and back position of merozoite as determined in the DIC channel. See also Fig. S1D,E. Scale bars: 1 μm.

We have previously imaged merozoite actin during *P. falciparum* erythrocyte invasion (Angrisano et al., 2012; Riglar et al., 2011; Wong et al., 2011), showing a concentration of actin in a band at the parasite periphery, extending from the tight junction towards the rear of the parasite. Using 3D SIM, the concentration of actin at the tight junction appeared to be slightly posterior to the movement of the junction as the parasite invaded (Angrisano et al., 2012). Using the current workflow, actin labelling similarly showed a consistent peak of intensity in the approximate region of the tight junction (Fig. 3A; Fig. S1D; *n*=19), varying in its position from distinctly behind the RON4-demarked tight junction (e.g. Fig. 3A, parasites 4 and 8) to overlapping with the junction (e.g. Fig. 3A, parasites 15 and 16). In almost all images analysed, actin fluorescence intensity was at least 50% of peak values at some position within the junction. Aligning the full range of parasites imaged demonstrated a tendency for parasites that are later in the invasion process to show higher levels of actin staining directly within the RON4-demarked junction (Fig. 3A, parasites 12–9). The bias in later stages may be explained by the steeper curvature of the parasite at its apex. In addition, ∼50% of parasites imaged showed strong apical actin labelling. These observations, although inconclusive with respect to the precise junction overlap and the possible cause for variable apical actin labelling, are consistent with the actomyosin traction force being at, or directly adjacent to, the tight junction, in line with recent reports from *T. gondii* (Bichet et al., 2014).

The role of aldolase as a necessary link between motor force and the host cell has recently come under renewed scrutiny, seeming to be superfluous to motor activity (Shen and Sibley, 2014). Longitudinal intensity profiling of the distribution of aldolase in the invading merozoite showed strikingly similar features to that of actin (Fig. 3B; Fig. S1E; *n*=19). Foremost, peak aldolase fluorescence was consistently found just rearward of the RON4-demarked tight junction (e.g. Fig. 3B, parasites 6 and 10) and, in addition, aldolase fluorescence within the tight junction was regularly at least 50% of peak intensity levels. Of note, some parasites displayed a peripheral labelling pattern for aldolase (e.g. Fig. 3B, parasites 14 and 16) as has been described previously, albeit with conflicting evidence, in extracellular *T. gondii* (Pomel et al., 2008; Starnes et al., 2009). Combined, these data are consistent with aldolase being present at the site of motor activity and junction formation, irrespective of whether aldolase is a necessary link to the motor or a recruited component of the energy-production machinery required for invasion.

### MTRAP localisation during invasion questions its proposed role in invasion

Under the current actomyosin motor model, filamentous actin links through to proteins of the thrombospondin-related anonymous protein (TRAP) family, which act as key traction mediators in both motility (gliding in sporozoites and ookinetes) and invasion [for a review see Baum et al. (2008)]. In the case of the merozoite this is thought to utilise the stage-specific merozoite TRAP orthologue MTRAP (Baum et al., 2006b). This family of proteins shares various features including the presence of adhesive extracellular domains that might assist in attachment to the host cell and, thus, act as a traction point during motility and invasion. In support of this model, MTRAP has recently been reported to interact with semaphorin 7A on the erythrocyte surface through its extra-parasitic thrombospondin repeat (TSR) domains (Bartholdson et al., 2012). Given this putative role in transmitting traction force, a key prediction of the motor model would be that MTRAP should localise to the junction during invasion.

**Fig. 4.**
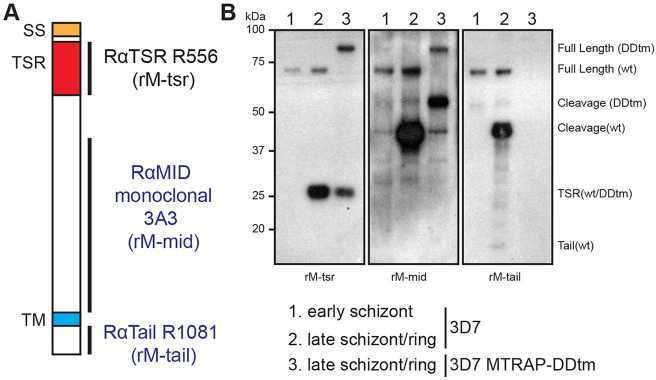
**MTRAP organisation and profile of domain-specific antibodies.** (A) MTRAP consists of a signal sequence (SS, orange), two thrombospondin repeat domains (TSR, red) and a single transmembrane domain (TM, blue), with a short C-terminal cytoplasmic tail. In order to track the various cleavage products of MTRAP we generated a rabbit monoclonal antibody (3A3) against the unstructured mid domain (rM-mid) and rabbit antiserum (R1081) against the short C-terminal tail (rM-tail). These antibodies complement the previously published MTRAP TSR (R556) antiserum (rM-tsr). (B) Analysis of 3D7 schizont and mixed schizont and ring (schizont/ring)-stage parasites along with schizont/ring-stage parasites expressing a DDtm-tagged version of MTRAP (MTRAP_DDtm_), were used to test antibody recognition. Immunoblot analysis labelled with rM-tsr antiserum identified full-length wild-type (wt) MTRAP at ∼75 kDa, full-length MTRAP_DDtm­_ at ∼90 kDa, and the cleaved MTRAP TSR domain at ∼27 kDa in both late schizont/ring samples as expected. rM-mid antibodies also recognised the full-length products, demonstrating its ability to specifically label MTRAP. Additionally, rM-mid recognised at least one product at ∼44 kDa in 3D7 samples. A corresponding band running at ∼55 kDa in MTRAP_DDtm_ parasites confirms that this is a cleavage product of MTRAP, which maintains its C-terminal tail. In some samples a second cleavage product running at ∼38 kDa was also seen (data not shown). rM-tail antiserum recognised the same full-length and cleavage products in 3D7 samples. Additionally, a faint band was consistently labelled running at ∼15 kDa in samples involving early ring stage parasites, but was absent in schizont stage parasites. We believe this corresponds to the MTRAP C-terminal tail stub following intramembrane cleavage. rM-tail antiserum was unable to label C-terminally-tagged versions of MTRAP.

**Fig. 5.**
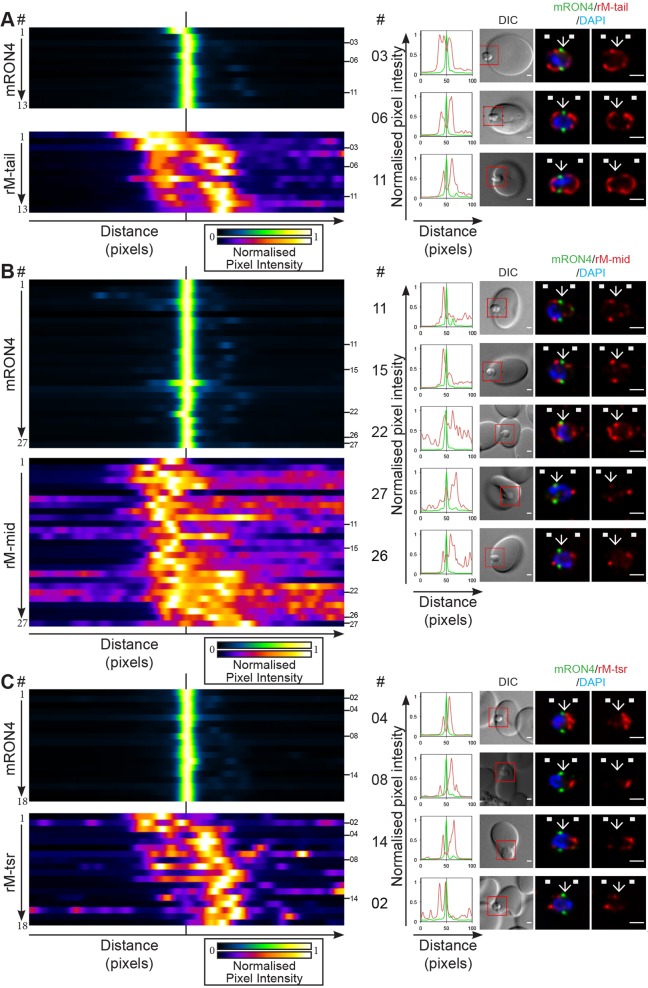
**Longitudinal intensity profiling identifies unexpected localisation profiles for MTRAP during merozoite invasion.** (A-C) Heatmaps, normalised intensity plots and single-slice images of example merozoites for the longitudinal intensity profiling of mRON4 (green) versus (A) rM-tail (red), (B) rM-mid (red) or (C) rM-tsr (red). Scale bars: 1 μm. The boxed regions in DIC images are shown magnified to the right. Arrows indicate the position of the RON4-labelled tight junction. White boxes denote approximate front and back position of merozoite as determined in the DIC channel. See also Fig. S2A–C. Scale bars: 1 μm.

One challenge in the assessment of MTRAP localisation during invasion is that it undergoes several processing events during maturation, resulting in cleavage of the extracellular TSR domain from the stub of cytoplasmic tail at or prior to invasion (Baum et al., 2006b). Towards assessing each component, we generated rabbit antisera against the C-terminal tail of MTRAP and a rabbit monoclonal antibody against a peptide spanning an unstructured region in the extracellular domain downstream of the TSR region, termed the mid domain (Fig. 4A). Combined with rabbit antisera generated against the MTRAP TSR domain (Baum et al., 2006b), these sera enabled labelling and distinction of the various MTRAP species (Fig. 4B), which, along with RON4 labelling, revealed via longitudinal intensity profiling several significant insights into MTRAP biology (Fig. 5A–C; Fig. S2A–C).

MTRAP tail antisera consistently labelled the merozoite surface (e.g. Fig. 5A, parasites 3, 6 and 11; Fig. S2A; *n*=13). Strong apical labelling was also regularly seen using this antiserum (e.g. Fig. 5A, parasite 6). Both distributions are consistent with MTRAP micronemal localisation and release onto the merozoite surface during the invasion period (Baum et al., 2006b). Of note, however, MTRAP labelling intensity was often at a local minimum within the RON4-demarked tight junction. Labelling was also commonly absent in the region immediately to the rear of the merozoite from the tight junction, which is where actin and aldolase were also regularly concentrated. This latter observation could suggest either an absence of MTRAP within the tight junction zone, caused by either active or passive exclusion, the presence of epitope masking similar to that observed with AMA1 IF9 labelling (see above), or issues with general antibody accessibility within the junction region of the periplasmic space. To investigate this latter possibility we tested the inner membrane complex and plasma membrane-anchored protein GAP45, which in developing merozoites shows a consistent labelling surrounding the parasite and would be expected to localise within the same periplasmic space as the MTRAP tail (Baum et al., 2006b). Although GAP45 intensity commonly dropped slightly within the region of the tight junction, intensity was regularly well above 50% of its peak in this region (Fig. S3A; *n*=15). Combined with the regular observation of fluorescence within the junction region of parasites labelled with actin and aldolase antisera, both of which are periplasmic proteins, we concluded that antibody access to the inner face of the junction region is unlikely to be the sole cause of fluorescence minima in the region. It remains possible that the MTRAP tail still experiences epitope masking at the tight junction, since the polyclonal serum was raised against only the short C-terminal tail peptide, which might have given rise to a markedly reduced repertoire of potential epitopes. Evidence for this limitation is seen when the endogenous MTRAP protein is tagged at its C-terminus, which renders the protein unrecognisable by this antiserum (Fig. 4B). Nonetheless, the balance of data presented here runs contrary to our expectations of MTRAP concentration solely at the junction.

Given our inability to come to a clear conclusion from investigation of MTRAP tail localisation, we next investigated the localisation of the MTRAP mid domain during invasion (Fig. 5B; Fig. S2B; *n*=27). The MTRAP mid domain appeared to localise in a punctate or dappled fashion around the merozoite surface in addition to commonly strong apical fluorescence, which was most likely associated with unreleased micronemes. Punctate background fluorescence was often visible within erythrocytes and manifested as high and variable baseline fluorescence in longitudinal intensity profiles, in particular at the front end (right-hand side) of the profiles (e.g. Fig. 5B, parasites 11, 22, 26). Close inspection of intensity profiles, however, did highlight the presence of high-intensity labelling just rearward of, and often directly within, the RON4-labelled tight junction in many (e.g. Fig. 5B, parasites 11, 15, 22, 27) but not all (e.g. Fig. 5B, parasite 26) parasites. An explanation for the differences in restricted versus more diffuse localisation between the mid domain and tail antibodies, respectively, might be related to the predicted proteolytic cleavage and retention of the tail stub post-MTRAP function during invasion.

To help resolve this, we investigated the localisation of the TSR domain (Fig. 5C; Fig. S2C; *n*=18), the expected functional adhesive domain of MTRAP (Bartholdson et al., 2012). Labelling was most commonly found only at the apical tip of the parasite (e.g. Fig. 5C, parasites 4, 8) with small patches of fluorescence at times also present in distinct areas of the parasite periphery or internally within the merozoite (e.g. Fig. 5C, parasites 14, 2). These observations were confirmed in the longitudinal intensity profiles, which most commonly showed only a strong apical peak within the parasite at varying distances from the RON4-labelled junction corresponding to an individual parasite's progression through invasion. Strikingly, TSR signal intensity was commonly almost completely absent within the zone of the tight junction and rarely present rearward from the junction. These results are entirely contrary to predictions given the presumed adhesive role of the MTRAP TSR domain and its demonstrated interaction with the erythrocyte surface receptor semaphorin 7A (Bartholdson et al., 2012). Furthermore, given the polyclonal nature of MTRAP TSR antisera and the clear ability of antibodies to access the junction, as demonstrated with the AMA1 polyclonal serum, we do not believe this to be an issue of epitope masking.

### Spatiotemporal analysis of MTRAP cleavage

Given the surprising lack of mid-invasion surface labelling by MTRAP TSR, but labelling by the MTRAP mid and tail domains, we investigated the spatiotemporal processing of MTRAP before and during invasion using a complementary approach towards the final assessment of its functional role in invasion. MTRAP has previously been shown to undergo processing within its extracellular domain by immunoblot (Baum et al., 2006b). The protein also possesses an intramembrane rhomboid protease cleavage recognition site, which can be cleaved by PfRhom4 in a heterologous assay (Baker et al., 2006). Like other TRAP family proteins or invasion ligands, our current hypothesis suggests that MTRAP is shed from the merozoite surface during invasion (O'Donnell and Blackman, 2005). The N-terminal TSR domain of MTRAP is abundant within culture supernatant, in support of its cleavage and release post invasion (Baum et al., 2006b).

**Fig. 6.**
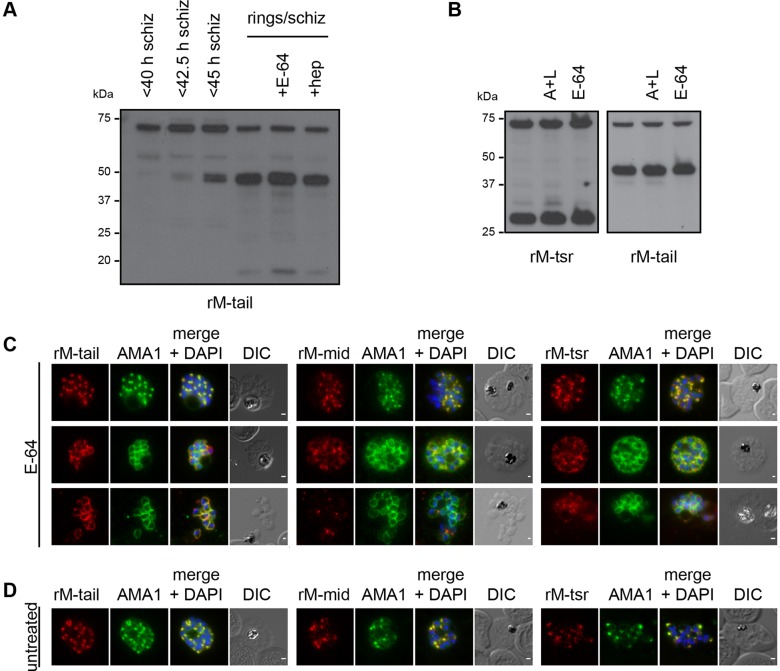
**MTRAP is cleaved irrespective of invasion or egress from the schizont.** (A) Immunoblot analysis of a timecourse of tightly synchronised schizonts and early ring samples grown in the presence or absence of inhibitors of egress (E-64) or invasion [heparin (hep)]. Labelling with rM-tail antibodies labels full-length MTRAP (∼75 kDa) and cleavage products (∼38/44 kDa), including the tail stub (∼15 kDa). (B) Immunoblot analysis of tightly synchronised late-schizont- and early-ring-stage parasites grown in the presence or absence of inhibitors of egress [antipain and leupeptin (A+L)] or E-64. Labelling with rM-tsr antisera identifies full-length MTRAP (∼75 kDa) and the cleaved TSR domain (∼25 kDa). rM-tail labelling identified products as in A. (C,D) Immunofluorescence analysis of very late-stage schizonts (C) treated with E-64 to prevent egress or (D) from untreated culture, co-labelled with mAMA1 (1F9) monoclonal antibodies (green) and rM-tail (left), rM-mid (middle) or rM-tsr (right) antibodies (red in respective images). Scale bars: 1 μm.

Different MTRAP antibodies consistently recognised a distinct set of MTRAP fragments (Fig. 4B): MTRAP TSR antiserum labelled full-length MTRAP, which runs on SDS-PAGE gels at ∼75 kDa, and the N-terminal fragment post cleavage, which runs at ∼27 kDa; MTRAP mid domain antibodies also recognised full-length MTRAP, along with two cleaved fragments running at ∼38 and ∼44 kDa; MTRAP tail antiserum recognised the same species as the mid domain antibodies, with the addition of a small band that ran at ∼15 kDa, which corresponds to the cleaved tail stub –although the latter species was never detected in high abundance, perhaps suggestive of fast degradation rates or low percentage retention during SDS-PAGE and immunoblot analysis. To investigate the cleavage of MTRAP during the lifecycle we performed a parasite developmental timecourse, harvesting tightly synchronised parasites either as <40 h, <42.5 h, <45 h post-invasion schizonts or following egress and reinvasion of a significant proportion of parasites (Fig. 6A, lanes 1–4). Immunoblot analysis using rabbit MTRAP tail antiserum showed the progressive appearance of ∼44 kDa cleavage products during schizogony (Fig. 6A, lanes 2 and 3), and of cleaved MTRAP tail in the ring-schizont mixed population (Fig. 6A, lane 4). The early appearance of ∼44 kDa cleavage products in schizont samples was unexpected as it would indicate a separation of the N-terminal TSR domain from the remainder of MTRAP, a process previously presumed to occur during invasion in a shedding-type event.

The possibility remained that the cleavage detected might derive from breakthrough or fast-growing parasites that had already undergone egress and invasion. To determine the dependency of cleavage on merozoite invasion and rupture, we treated parasites that had been synchronised simultaneously with those above with either the carbohydrate heparin, which is an inhibitor of merozoite invasion (Boyle et al., 2010a), or the cysteine protease inhibitor E-64, which is an inhibitor of merozoite egress (Salmon et al., 2001). These parasites were allowed to grow and rupture in the presence of the respective drugs before being harvested simultaneously with mixed ring-schizont samples (Fig. 6A, lanes 5 and 6). Strikingly, the presence of all major MTRAP cleavage products in drug-treated samples suggested that MTRAP cleavage occurred independently of both invasion and schizont rupture. Although it remains possible that MTRAP cleavage was occurring aberrantly from the surface of the heparin-inhibited merozoites that remained in the medium (Fig. 6A, lane 6), only low levels of breakthrough invasion were seen following E-64 treatment (data not shown), thus, making it highly unlikely that a similar degree of cleavage could be attributed to only those parasites escaping E-64 inhibition (Fig. 6A, lane 5).

Given this unexpected early cleavage of the N-terminal, TSR-containing domain from the rest of the MTRAP protein, we further investigated processing using E-64 and treatment with a combination of the protease inhibitors antipain and leupeptin, which together act to block egress (Wickham et al., 2003). As before, inhibition of egress had no effect on the cleavage of the TSR domain, as measured by release of the ∼27 kDa TSR fragment labelled using rabbit antiserum raised against the TSR domain and by the presence of the ∼44 kDa MTRAP fragment labelled using rabbit antiserum raised against the C-terminal tail (Fig. 6B).

Although our results suggested that cleavage could occur independently of invasion, contact with erythrocytes and even correct egress, we wanted to investigate whether the model for shedding was still valid by determining whether cleavage occurred only after microneme release and exposure of MTRAP on the merozoite surface. Owing to the difficulties in obtaining sufficient numbers of appropriately late schizonts for rigorous analysis, we synchronised parasites to a ∼4 h window, treated late schizonts with E-64 and allowed them to progress past their expected time of rupture. The resulting samples contained parasites ranging from late schizonts to the membrane-confined sacs of merozoites typical of E-64 treatment. Immunofluorescence analysis of these parasites, following their fixation on glass slides using methanol, was used to co-label AMA1 and MTRAP using the various MTRAP antisera (Fig. 6C). E-64-treated samples consistently showed evidence of microneme secretion in later stage parasites. In many parasites, AMA1 labelling localised to the merozoite surface (Fig. 6C), although earlier stage schizonts that still showed the punctate fluorescence associated with unreleased micronemes were also present within the samples (Fig. 6C, top panels). Co-labelling of the MTRAP tail (Fig. 6C, left panels) showed progressively increased labelling associated with the merozoite surface in parasites displaying partial and full AMA1 surface distribution. Conversely, MTRAP mid (Fig. 6C, middle panels) and TSR (Fig. 6C, right panels) both showed dispersed labelling in E-64-treated schizonts and evidence of reduced labelling and increased dispersal of signal in parasites that appeared to have at least partially burst from their surrounding membranes (Fig. 6C, bottom panels).

To relate these results to untreated parasites, we next imaged tightly synchronised schizonts co-labelled with AMA1 and MTRAP antibodies (Fig. 6D). Although the vast majority of parasites in these samples showed no evidence of microneme secretion, a small percentage commonly showed evidence of partial AMA1 redistribution to the merozoite surface, indicative of microneme release beginning prior to egress from the schizont. This is consistent with the timing of AMA1 secretion following reversible treatment of parasites with the PfPKG inhibitor Compound 1 (C1) (Collins et al., 2013b). We did not observe any parasites showing complete AMA1 redistribution or any clear MTRAP redistribution to the merozoite surface, suggesting that this phenotype might be specific to parasites arrested from egress using E-64. These results are, therefore, consistent with both the TSR and mid domains of MTRAP being released from the merozoite surface following microneme secretion, but in a manner independent of invasion or egress from the schizont. It remains unclear whether MTRAP, like AMA1, is released onto the merozoite surface just prior to egress or if MTRAP and AMA1 reside in distinct populations of micronemes.

**Fig. 7.**
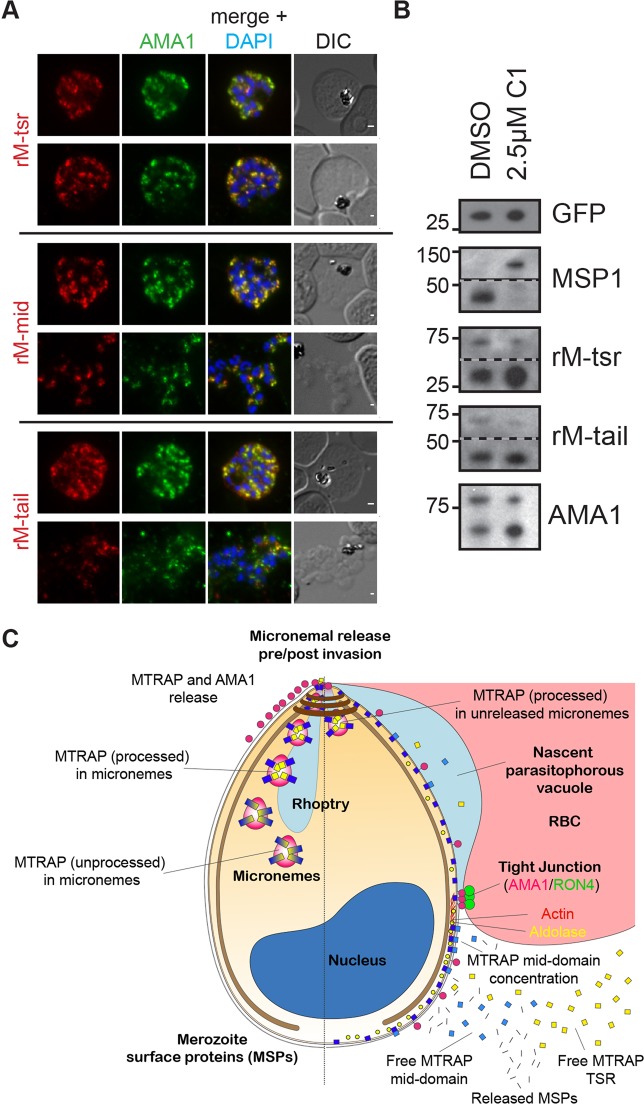
**MTRAP is cleaved within the micronemes.** (A) Immunofluorescence analysis of Compound 1 (C1)-treated schizonts (versus DMSO control) co-labelled with mAMA1 (green) and rM-tsr (top), rM-mid (middle) or rM-tail (bottom) (red in respective images) antibodies. Scale bars: 1 μm. (B) Immunoblot analysis of tightly synchronised late-stage schizont and early ring parasites labelled for GFP (loading control), MSP1 (C1 treatment control), rM-TSR, rM-tail and rAMA1. (C) Schematic of pre- (left half) and post- (right half) invasion merozoites, showing the predominant distribution of each protein examined as part of this study. It should be noted that all proteins show a degree of variability in labelling distribution across merozoites.

Finally, to investigate whether MTRAP cleavage could occur prior to microneme secretion, we arrested tightly synchronised parasites prior to microneme secretion using C1 (Fig. 7) (Collins et al., 2013b). Co-labelling with AMA1 and MTRAP antibodies confirmed that parasites treated in this manner retained only punctate localisations of both proteins, even in cases in which merozoites appeared to have burst from the membranes surrounding the schizont (Fig. 7A). Immunoblot analysis of saponin-lysed parasite samples was then undertaken to assess the cleavage state of MTRAP (Fig. 7B). The activity of C1 was confirmed by immunoblot, which showed cleavage of MSP1 to form the MSP1-42 cleavage product in DMSO-treated control parasites but not in parasites treated with 2.5 μM C1. Surprisingly, despite the lack of microneme secretion, there was clear evidence of MTRAP cleavage to release the 27 kDa TSR-containing fragment, as recognised by the antiserum raised against the MTRAP TSR domain, and the ∼44 kDa product labelled by antiserum raised against the MTRAP C-terminal tail (Fig. 7B). Similarly, AMA1 prodomain cleavage to form the 66 kDa product occurred independently of microneme secretion (Fig. 7B).

Taken together, these results suggest that the TSR domain of MTRAP is cleaved from the remainder of the protein prior to secretion onto the merozoite surface (Fig. 7C). Thus, as uncovered by our imaging approach and followed up with biochemical and imaging assessment of MTRAP distribution, these results strongly call into question our current MTRAP-dependent model for merozoite invasion.

## DISCUSSION

One of the key challenges of fluorescence microscopy and its application to cell biology is that of subjectivity. How do we assess the reliability of images, acquired with all possible rigor (Waters and Wittmann, 2014), in the support or rejection of a hypothesis without bias? For single-molecule imaging, there is already a large discipline of statistical approaches for assessing molecule distribution that can be used to rigorously test the localisation of different proteins (Sauer, 2013). For conventional imaging, however, although there is a general understanding that unbiased approaches are important there are few guidelines as to how many images are required or what analytical methods should be used to back up a scientific statement. For example, for an immunoblot or protein electrophoresis gel, it is widely recognised that appropriate replication and comparative densitometry (with a good negative control) provide a simple way of making an otherwise singular result more robust. But for a live cellular process such approaches are ill defined and many studies continue to be published in which a hypothesis stands or falls backed up by a singular striking image of a cell. In addition, it is obvious that in a biological system no single image could possibly encapsulate all eventualities. These issues are particularly relevant when the cell of interest is only ∼1 μm in length, as in the case of the *Plasmodium* parasite, and the biological process is hard to capture, as with this parasite's entry into the human erythrocyte en route to causing malaria disease.

Here we have tried to address image bias and biological variability in the application of 3D immunofluorescence microscopy to merozoite invasion. Using recent advances in methods to capture and image the process of invasion of erythrocytes (Boyle et al., 2010b; Riglar et al., 2011), we have developed a workflow that creates a standardisation of imaging events permitting comparisons between samples to assess the distribution of various test proteins during invasion. Although at the resolution limits of deconvolution microscopy, our goal has been to provide a workflow that is possible to replicate using best-practice fluorescence imaging on a conventional widefield microscope. The imaging requires no more than the ability to make a 3D *z*-stack and access to good antibodies (Bordeaux et al., 2010). Using this approach, which we refer to as longitudinal intensity profiling, we assessed whether the fluorescence signals associated with labelled proteins on the invading merozoite fit with the hypothesized function of these proteins.

Our approach makes several striking observations about the process of merozoite invasion and the application of fluorescence imaging. Most sobering is the recognition that multiple antibodies or epitopes (such as in polyclonal antiserum) are required in assessing localisation in order to avoid potential problems with singular epitopes being masked or poorly accessible under certain fixation conditions. This is clearly demonstrated with the distribution of the invasion protein AMA1. Whereas previous data presented dual viewpoints as to the presence (Alexander et al., 2005; Riglar et al., 2011) or absence (Giovannini et al., 2011) of AMA1 at the tight junction during apicomplexan parasite entry into host cells, here we show that both scenarios can be explained, the latter being the result of masking of the antigen epitope targeted by the monoclonal antibody (Coley et al., 2007). This is likely to be the case for monoclonal antibodies used for imaging *Toxoplasma* AMA1 during tachyzoite invasion (Giovannini et al., 2011). Thus, we believe that our approach clearly demonstrates that AMA1 does track the junction; however, the longitudinal profile also shows that a large proportion of AMA1 is also carried within the apical complex (probably in unsecreted micronemes) or is distributed variably on the cell surface of the invading parasite (Fig. 2, Fig. 7C).

Applying the workflow to the imaging of motor components also reveals several insights. Foremost, actin concentrates and aldolase is present at or immediately behind the junction as the parasite enters (Fig. 3, Fig. 7C). Although the view that aldolase plays no force transduction role in the mechanics of parasite motility and invasion is now gaining acceptance (Shen and Sibley, 2014), it is entirely feasible that aldolase may nonetheless localise with the junction as the parasite invades. That is, energy production may be concentrated around the gliding motor during invasion, a concept that is consistent with – albeit contested (Starnes et al., 2009) – evidence for relocalisation of the glycolytic enzymes at this time (Pomel et al., 2008).

Although the localisation of actin and aldolase might fit with an evolving model of gliding motor organisation, our data on MTRAP leave current understanding much more unresolved. The TRAP-like proteins are envisaged to provide an unbroken linkage between the intracellular actomyosin motor and the extracellular milieu (Baum et al., 2008). The absence of the adhesive TSR domain of MTRAP at the junction (Fig. 5) and accumulating evidence that it is preprocessed from the cytoplasmic domain well before invasion, probably prior to the egress of merozoites from the infected erythrocyte (Figs 6 and 7), question whether it can form an unbroken linkage with the internal gliding motor as originally envisaged (Baum et al., 2006b) (Fig. 7C). Indeed, our own efforts at reciprocally immunoprecipitating the MTRAP head (TSR) and cytoplasmic tail were unsuccessful (data not shown), suggesting that, post processing, the two parts of MTRAP might not even stay together. Recent evidence suggests that the shedding of the extracellular domain of liver stage TRAP is essential for both gliding motility and host infectivity (Ejigiri et al., 2012). Thus, what might be emerging is a sense that it is the processing and release of adhesive domains that either provides the necessary signals to the parasite for motility and invasion or that might even provide a substrate for other adhesive ligands to bind. Further exploration of the function of adhesive domains in providing the conduit for motor traction will be important in resolving TRAP-like protein function (Hegge et al., 2010; Hellmann et al., 2013; Münter et al., 2009).

Thus, we now believe it is entirely plausible that MTRAP plays no direct role in mediating invasion of the erythrocyte but might instead function in a signalling role, either with TSR binding to the erythrocyte surface (Bartholdson et al., 2012) or with rupture priming the erythrocyte at invasion, or some other binding event that does not require linkage to the junction or motor machinery. Our previous attempts to knock out MTRAP failed by conventional double crossover recombination (Baum et al., 2006b). Additional attempts to knock down MTRAP levels using various conditional knockout systems, including both FKBP-based (Armstrong and Goldberg, 2007) and DHFR-based (Muralidharan et al., 2011) destabilisation domains, were unsuccessful (Fig. S3B–E). In each case, while integration of the respective domains occurred in almost all parasites within two integration cycles, no evidence for regulation of protein levels was seen. With recent advances in conditional genetic systems (Collins et al., 2013a; Yap et al., 2014) it might yet be possible to make a conditional knockout to explore the function of MTRAP directly and test whether its role relates to the mechanics of invasion or some other signalling event. Future work in this area is clearly of interest.

In summary, we present a coherent workflow for assessing the localisation of proteins during the transient process of *Plasmodium* merozoite invasion of the human erythrocyte. This approach, termed longitudinal intensity profiling, might be applicable to multiple cellular systems beyond parasite invasion. Our data highlight the importance of antibody selection and multiple epitope targeting in generating unbiased fluorescence imaging data to test hypotheses. Absence of signal can still be explained by alternative hypotheses (such as epitope masking), in which case our data also stress the need for non-imaging methodologies to back up hypotheses of function in cases where protein localisation is unclear.

## MATERIALS AND METHODS

### Parasite tissue culture and merozoite isolation

*P. falciparum* D10-PfM3′ asexual stage parasites (O'Donnell et al., 2001) were maintained using standard culture conditions in human O+ erythrocytes (Australian Red Cross Blood Bank, South Melbourne, Australia). Culture medium was RPMI-HEPES with 0.18% NaHCO_3_ and 0.5% Albumax II (Life Technologies) or 10% pooled human serum from unexposed donors (Australian Red Cross Blood Bank). Parasites were synchronised using a combination of sorbitol (Sigma-Aldrich) and heparin (Pfizer) synchronisation methods (Boyle et al., 2010a; Lambros and Vanderberg, 1979).

Purified invasive merozoites were isolated and fixed during invasion as previously described (Riglar and Baum, 2013). Briefly, tightly synchronised late stage schizonts (>40 h) were isolated from ∼90 ml culture by magnet purification (MACS Miltenyi Biotech) and treated with 10 μM trans-epoxysuccinyl-L-leucylamido(4-guanidino)butane (E-64; Sigma-Aldrich) for 6–8 h in ∼30 ml culture medium before being pelleted by centrifugation (1900 ***g***, 5 min), resuspended in a small amount of serum-free medium (typically ∼0.75 ml), filtered through a 1.2 μm, 32 mm syringe filter (Sartorius Stedim Biotech) and mixed immediately with fresh human erythrocytes (typically ∼15 μl of packed erythrocytes washed and resuspended at 50% in serum-free culture medium) at 37°C with shaking. After 2 min, invading merozoites were fixed with 4% paraformaldehyde (ProSciTech) containing 0.0075% glutaraldehyde (ProSciTech) in PBS for 30 min at room temperature, followed by permeabilisation with 0.1% Triton X-100 (Bio-Rad) for 10 min and blocking overnight or longer at 4°C with filtered 3% bovine serum albumin (Sigma-Aldrich) in PBS (blocking solution).

For schizont stage imaging, thin smears were taken directly from culture and air dried before being fixed in 100% methanol at −20°C for 30 s. After drying, slides were blocked for at least 30 min at room temperature with blocking solution.

For cleavage analysis, synchronised parasites were treated with a combination of antipain and leupeptin (10 μg/ml each; Sigma-Aldrich), E-64 as described above, or 2.5 μM pyrrole 4-[2-(4-fluorophenyl)-5-(1-methylpiperidin-4-yl)-1H-pyrrol-3-yl] pyridine (Compound 1; SYNthesis Med Chem) (Collins et al., 2013b).

### Immunofluorescence detection of proteins

Blocked invading parasites or schizont smears were incubated, in solution or on slide, respectively, with primary antibodies diluted in blocking solution for 1 h at room temperature. The following dilutions were used: mouse anti-RON4 (Richard et al., 2010), 1:500; rabbit anti-RON4 (Richard et al., 2010), 1:250; mouse anti-AMA1 1F9 (Coley et al., 2001), 1:200; rabbit anti-AMA1 R190 (Healer et al., 2002), 1:200; rabbit anti-MTRAP TSR (Baum et al., 2006b), 1:50–1:100; rabbit anti-MTRAP mid (this study), 1:25; rabbit anti-MTRAP tail (this study), 1:250–1:500; rabbit anti-actin (Angrisano et al., 2012), 1:200; rabbit anti-aldolase (Baum et al., 2006b), 1:200; rabbit anti-GAP45 (Baum et al., 2006b), 1:200. Following primary incubation, samples were washed twice for 5 min each with PBS, incubated for 1 h at room temperature with Alexa 488- or Alexa 594-conjugated secondary antibodies raised against the appropriate species, diluted 1:500 in blocking solution (Life Technologies), then washed three times for 5 min each with PBS. Invading merozoite samples were settled for 30 min at room temperature onto high-performance #1.5-grade coverslips (Carl Zeiss), preflamed and coated with a 1% polyethyleneimine solution (Sigma-Aldrich). Coverslips were mounted in VectaShield (Vector Laboratories) with 0.1 ng/μl 4′,6-diamidino-2-phenylindole (DAPI; Life Technologies). Results shown are representative of multiple experiments with antibodies tested empirically on each day of use to control for batch-to-batch variation.

Imaging was performed on a DeltaVision Elite widefield microscope (Applied Precision) using a 100×, 1.4 NA Super-Plan APO oil-immersion objective (Olympus), InsightSSI solid-state illumination, and a CoolSnap HQ2 CCD camera (Photometrics). Filter sets used were DAPI [excitation (ex), 390/18 nm; emission (em), 435/48 nm], FITC (ex, 475/28; em, 525/48 nm) and Alexa Fluor 594 (ex, 575/25; em, 625/45 nm). Image stacks were captured well above and below the parasite with a *z*-step of 150 nm and deconvolution undertaken in the DeltaVision SoftWoRx software package.

Image processing was routinely performed using ImageJ (NIH). A custom ImageJ macro, the Figure Wizard (https://github.com/DrLachie/Figure_Wizard), was used extensively in creation of the final figure panels.

### Immunoblot detection of proteins

For immunoblot analysis, tightly synchronised parasites were harvested by centrifugation at the appropriate time, released from erythrocytes in 1.5 volumes of 0.15% saponin+cOmplete protease inhibitor (Roche Applied Biosciences) for 10 min on ice, washed with ice-cold PBS and solubilised in 2× SDS loading buffer. Proteins were separated on NuPAGE Novex 4–12% Bis-Tris gels with MES Buffer (Life Technologies) and transferred to 0.2 μm PVDF membrane using the iBlot system (Life Technologies). Membranes were blocked in PBS containing 5% (w/v) skimmed milk and 0.1% Tween 20 (Sigma-Aldrich) for 1 h at room temperature then incubated for 1 h with primary antisera diluted as follows in PBS containing 1% (w/v) skimmed milk and 0.1% Tween 20: affinity-purified rabbit anti-MTRAP TSR (rM-tsr), 1:500 (Baum et al., 2006b); rabbit anti-MTRAP mid monoclonal (rM-mid), 1:25 (this study); rabbit anti-MTRAP tail (rM-tail), 1:1000 (this study); mouse anti-GFP monoclonal, 1:1000 (clones 7.1/13.1, Roche Applied Biosciences); and anti-MSP1 (Cooper et al., 1992), 1:1000. After washing, signal was detected with appropriate IgG horseradish peroxidase (HRP) conjugates (Merck Millipore), followed by further washes and chemiluminescence detection (ECL, Amersham Biosciences).

### Image acquisition and processing work flow for analysing multiple invasion events

Invading free merozoites were prepared for indirect immunofluorescence assay and imaged as described above. In each case the tight junction-associated protein RON4 (Richard et al., 2010) was included as one of two primary antibodies. Parasites were selected for imaging using three criteria: the presence of a clear ring of labelling with RON4 indicating a parasite close to the midpoint of invasion; invasion occurring approximately planar with the microscope *x-y* plane; and the absence of confounding merozoites in the vicinity. The labelling of non-RON4 comparison antibodies was not taken into account. To analyse the distribution of antibody labelling along the length of a merozoite, images were rotationally aligned in the *x-y* plane (Fig. 1Bi) and pixel intensity profiles were generated (Fig. 1Bii) in MetaMorph image analysis software (Molecular Devices). Values were recorded from a max intensity line profile with a line width of 45 pixels that was taken for 50 pixels along the long axis in each direction from the brightest intensity point in the RON4 channel. This analysis was repeated for all *z*-positions, a process that adjusts for deviation from an *x-y* planar invasion vector (i.e. where invasion was tilted in the *z*-axis). Slices in which the profile spuriously centred to a point outside of the bounds, as defined from the DIC image, of the merozoite (e.g. due to background fluorescence) were discarded automatically. The resulting values were then summed for the whole *z*-stack and normalised to the maximum value across each parasite, resulting in a single average linear representation for the 3D longitudinal labelling distribution across each merozoite (Fig. 1Biii). Once generated, images were then assembled in a mock-temporal fashion, i.e. assigning parasites as being early through late in the process of invasion as determined by the position of the junction relative to the apex or base of the merozoite. This mock temporal distribution was displayed using a heatmap to present all available data for a given antibody collectively. MetaMorph journals used for longitudinal intensity profiling are available at https://github.com/DrLachie/Longitudinal_Intensity_Profiler.

### MTRAP antiserum

A synthetic peptide encompassing the C-terminal residues of the MTRAP protein, N-CYFLRKEKTEKVVQEETKEENFEVMFNDDALKGKDNKAMDEEEFWALE-C, was used to generate rabbit polyclonal serum (GenScript). A recombinant protein encompassing valine (residue 95) to glutamic acid (residue 299) was generated by PCR amplification from *P. falciparum* genomic DNA, cloned by restriction enzyme digestion into a *Bam*HI- and *Xho*I-digested expression vector (pGEX-4T-1; GE Healthcare Biosciences) and expressed as a recombinant protein in *Escherichia coli* (strain BL21). The protein was purified over glutathione agarose (Sigma-Aldrich) using conventional methods (Baum et al., 2006b). The purified protein was used to immunise rabbits and generate both polyclonal and a monoclonal antibody (clone 3A3; Walter and Eliza Hall Monoclonal Facility, Bundoora, VIC, Australia).

### Vector construction and generation of destabilisation domain mutations and their analysis

PCR primers and vector maps are available on request. A derivative of the FKBP destabilisation domain, DDtm (Chu et al., 2008), and a DHFR degradation domain, DDD (Iwamoto et al., 2010), both of which have previously been used to successfully regulate proteins in *P. falciparum* (Dvorin et al., 2010; Muralidharan et al., 2011), were generated as C-terminal fusions of MTRAP. Fragments of either DDD [derived by PCR from the published vector (Muralidharan et al., 2011)] or DDtm [obtained as a synthetic DNA construct (Geneart, Life Technologies)] were inserted into the *Nhe*I and *Nco*I sites (replacing the triple HA tag) of vector pD3HA (Riglar et al., 2011). For the DDD vector, the DHFR selection cassette was replaced with that encoding blasticidin deaminase (Bsd), conferring resistance to blasticidin-S (Bs). Upstream of the inserted tag, an 813 bp 3′ flank ending at (but excluding) the MTRAP stop codon was PCR amplified and cloned in front of each domain to include a triple alanine linker in frame with either the DDtm or DDD sequence, generating plasmids pMTRAP-DDtm and pMTRAP-DDD. D10 and 3D7ΔRh3 parasites [the latter used for its integrated DHFR resistance (Baum et al., 2005), necessary to confer resistance to the trimethoprim (TMP) ligand] were transfected with respective DDtm and DDD plasmids using standard methods (Riglar et al., 2011), and selected with WR99210 or Bs, respectively, and grown in the presence of either 2 μM shield-1 (for DDtm) or 10 μM TMP (for DDD). Integration of 3′ tags was assessed after two cycles, at which point the regulation of MTRAP was tested. To test MTRAP regulation, parasites were tightly synchronised, washed at ring stage and grown in the presence or absence of the respective ligand. These conditions were maintained for two or three cycles for DDtm and DDD experiments, respectively, concurrently measuring parasitemia and taking protein samples for immunoblot analysis. 3D7ΔRh3 MTRAP-DDD parasites were also maintained in long-term standard culture in the absence of TMP to test MTRAP regulation.
